# Mutations in the GLA Gene and LysoGb3: Is It Really Anderson-Fabry Disease?

**DOI:** 10.3390/ijms19123726

**Published:** 2018-11-23

**Authors:** Giovanni Duro, Carmela Zizzo, Giuseppe Cammarata, Alessandro Burlina, Alberto Burlina, Giulia Polo, Simone Scalia, Roberta Oliveri, Serafina Sciarrino, Daniele Francofonte, Riccardo Alessandro, Antonio Pisani, Giuseppe Palladino, Rosa Napoletano, Maurizio Tenuta, Daniele Masarone, Giuseppe Limongelli, Eleonora Riccio, Andrea Frustaci, Cristina Chimenti, Claudio Ferri, Federico Pieruzzi, Maurizio Pieroni, Marco Spada, Cinzia Castana, Marina Caserta, Ines Monte, Margherita Stefania Rodolico, Sandro Feriozzi, Yuri Battaglia, Luisa Amico, Maria Angela Losi, Camillo Autore, Marco Lombardi, Carmine Zoccali, Alessandra Testa, Maurizio Postorino, Renzo Mignani, Elisabetta Zachara, Antonello Giordano, Paolo Colomba

**Affiliations:** 1 Institute of Biomedicine and Molecular Immunology “A. Monroy”, National Research Council, 90146 Palermo, Italy; giovanni.duro@ibim.cnr.it (G.D.); carmela.zizzo@ibim.cnr.it (C.Z.); giuseppe.cammarata@ibim.cnr.it (G.C.); simone.scalia@hotmail.it (S.S.); robertao.890@gmail.com (R.O.); sciarrino@ibim.cnr.it (S.S.); daniele.francofonte@ibim.cnr.it (D.F.); riccardo.alessandro@unipa.it (R.A.); 2 Neurological Unit, St Bassiano Hospital, 36061 Bassano del Grappa, Italy; alessandro.burlina@aulss7.veneto.it; 3 Division of Inherited Metabolic Diseases, Regional Center for Expanded Neonatal Screening Department of Women and Children’s Health, University Hospital of Padova, 35128 Padova, Italy; alberto.burlina@unipd.it (A.B.); giulia.polo@aopd.veneto.it (G.P.); 4 Department of Biopathology and Medical Biotechnologies—Section of Biology and Genetics, University of Palermo, 90133 Palermo, Italy; 5 Department of Public Health, Nephrology Unit, University of Naples “Federico II”, 80131 Naples, Italy; antonio.pisani13@gmail.com (A.P.); elyriccio@libero.it (E.R.); 6 Nephrology Unit, University Hospital “San Giovanni di Dio e Ruggi d’Aragona”, 84131 Salerno, Italy; giuseppe.palladino@sangiovannieruggi.it; 7 Neurology Unit, University Hospital “San Giovanni di Dio e Ruggi d’Aragona”, 84131 Salerno, Italy; napoletanorosa10@tiscali.it (R.N.); maurizio.tenuta@sangiovannieruggi.it (M.T.); 8 Division of Cardiology, Second University of Naples, Presidio Monaldi, 80131 Naples, Italy; danielemasarone@libero.it (D.M.); limongelligiuseppe@libero.it (G.L.); 9 Department of Cardiovascular, Respiratory, Nephrologic, Anesthesiologic and GeriatricSciences, Sapienza University, 00161 Rome, Italy; biocard@inmi.it (A.F.); cristina.chimenti@uniroma1.it (C.C.); 10 Nephrology Unit, Hospital of L’Aquila, 67100 L’Aquila, Italy; claudio.ferri@cc.univaq.it; 11 Nephrology Unit, Department of Medicine and Surgery, University of Milano-Bicocca, 20900 Monza, Italy; federico.pieruzzi@unimib.it; 12 Cardiovascular Department, San Donato Hospital, 52100 Arezzo, Italy; mauriziopieroni@yahoo.com; 13 Department of Pediatrics, Division of Metabolic Diseases, Turin University Hospital, 10126 Turin, Italy; marco.spada@unito.it; 14 Paediatric Hospital “G. Di Cristina”, ARNAS Civico, 90134 Palermo, Italy; cinziacastana@virgilio.it (C.C.); caserta_marina@hotmail.com (M.C.); 15 Cardiology Department Echocardiography Laboratory, Department of Cardiothoracic and Vascular, Policlinico Vittorio Emanuele, Catania University, 95124 Catania, Italy; inemonte@unict.it; 16 Institute of Neurological Sciences (ISN), National Research Council, 95126 Catania, Italy; margheritastefania.rodolico@cnr.it; 17 Nephrology and Dialysis Unit, Belcolle Hospital, 01100 Viterbo, Italy; sandro.feriozzi@tiscali.it; 18 Department of Specialized Medicine, Division of Nephrology and Dialysis, St. Anna Hospital-University, 44124 Ferrara, Italy; yuri.battaglia@ospfe.it; 19 Unit of Nephrology, Ospedali Riuniti Villa Sofia-Cervello, 90146 Palermo, Italy; amicoluisa@gmail.com; 20 Department of Advanced Medical Sciences, Federico II University of Naples, 80131 Naples, Italy; losi@unina.it; 21 Cardiology Unit, Clinical and Molecular Medicine Department, Faculty of Medicine and Psychology, Sapienza University of Rome, 00161 Rome, Italy; camillo.autore@uniroma1.it; 22 Nephrology and Dialysis Unit, Mugello Hospital, A.S. Toscana Centro, Borgo San Lorenzo, 50032 Firenze, Italy; lombardim@tin.it; 23 Institute of Clinical Physiology, Division of Nephrology, National Research Council, 89129 Reggio Calabria, Italy; carmine.zoccali@alice.it (C.Z.); atesta@ifc.cnr.it (A.T.); 24 Nephrology Unit, Grande Ospedale Metropolitano Reggio Calabria, 89124 Reggio Calabria, Italy; maurizio@postorino.eu; 25 Department of Nephrology, Infermi Hospital, 47923 Rimini, Italy; renzo.mignani@auslromagna.it; 26 Cardiac Arrhythmia Center and Cardiomyopathies Unit, San Camillo-Forlanini Hospital, 00152 Roma, Italy; e.zachara@scf.gov.it; 27 Department of Neurology, Guzzardi Hospital, 97019 Vittoria, Italy; antonellomaria.giordano@gmail.com

**Keywords:** Fabry disease, GLA gene, LysoGb3

## Abstract

Anderson-Fabry disease (FD) is a rare, progressive, multisystem storage disorder caused by the partial or total deficit of the lysosomal enzyme α-galactosidase A (α-Gal A). It is an X-linked, lysosomal enzymopathy due to mutations in the galactosidase alpha gene (GLA), encoding the α-Gal A. To date, more than 900 mutations in this gene have been described. In our laboratories, the study of genetic and enzymatic alterations related to FD was performed in about 17,000 subjects with a symptomatology referable to this disorder. The accumulation of globotriaosylsphingosine (LysoGb3) was determined in blood of positives. Exonic mutations in the GLA gene were detected in 471 patients (207 Probands and 264 relatives): 71.6% of mutations were associated with the classic phenotype, 19.8% were associated with the late-onset phenotype, and 8.6% of genetic variants were of unknown significance (GVUS). The accumulation of LysoGb3 was found in all male patients with a mutation responsible for classic or late-onset FD. LysoGb3 levels were consistent with the type of mutations and the symptomatology of patients. α-Gal A activity in these patients is absent or dramatically reduced. In recent years, confusion about the pathogenicity of some mutations led to an association between non-causative mutations and FD. Our study shows that the identification of FD patients is possible by associating clinical history, GLA gene analysis, α-Gal A assay, and blood accumulation of LysoGB3. In our experience, LysoGB3 can be considered a reliable marker, which is very useful to confirm the diagnosis of Fabry disease.

## 1. Introduction

Anderson-Fabry (or Fabry) disease (FD) (OMIM #301500) takes the name from the two dermatologists that independently described for the first time the pathology at the end of 1800 [1]. It is an X-linked, lysosomal enzymopathy caused by mutations in the GLA gene, encoding the α-galactosidase A (α-Gal A) [2]. FD is a sphingolipidosis that is considered rare, with a prevalence of 1:40,000. However, recent studies have showed that this disorder is much more widespread than is commonly believed [3,4].

Fabry disease is a lysosomal storage disorder due to a functional defect of the enzyme α-Gal A. This deficit is responsible for the alteration of the metabolism of some glycosphingolipids, mainly globotriaosylceramide (Gb3), which consequently build up in the lysosomes of different cellular types, mostly in vascular endothelium cells [5]. This results in systemic symptomatology with renal, cardiac, and cerebrovascular involvement, which may lead, at the fourth or fifth decade of life, to premature death [6]. First symptoms can occur at different ages, usually during childhood, and show different severity and clinical courses. The classic clinical manifestations of FD occur during pediatric age with lancinating acroparesthesias in the extremities. The autonomic nervous system impairment is responsible for alteration of sweating and thermoregulation disorder: during fever or physical exertion, FD patients can manifest typical crises of pain. Gastrointestinal pain, associated with diarrhea and sensation of abdominal fullness, is caused by the involvement of the vegetative local system. The skin in FD can manifest the typical angiokeratomas localized mainly in the swimsuit area or throughout the body (*angiokeratomas corporis diffusum*). Moreover, patients can have lymphedemas mostly in the lower limbs. The eye, like in many neuro-metabolic diseases, can show different signs such as the typical *cornea verticillata*, cataract, and telangiectasia of the conjunctiva. The function of renal cells is compromised by the storage of glycosphingolipids, leading to a progressive nephropathy that is one of the most critical manifestations of the disease. The hearth of FD patients frequently shows a left ventricular hypertrophy, seemingly idiopathic, and an impairment of valvular apparatus and intracardiac conduction. This involvement can cause various complications, such as cardiac failure or arrhythmic pathology, which can lead to death caused by myocardial infarction. Besides the impairment of the peripheral nervous system, FD patients can present an involvement of the brain with juvenile ischemic strokes and possible hemorrhagic episodes. In addition to the classic phenotype, atypical variants were described in patients with a milder symptomatology, an involvement of a single organ, and late-onset [7,8,9]. Due to the X-linked transmission of FD, female patients have long been considered only carriers. Conversely, they can develop significant signs and symptoms of the disease, even if the phenotype is generally less severe [10]. Furthermore, female patients usually show a variable symptomatology, because of the lyonization of the X-chromosome [11].

Nowadays the diagnosis of FD is still difficult, especially because its clinical manifestations overlap with the ones of other pathologies, and, for this reason, there is a wide possibility of differential diagnoses made by different medical specialists [12]. The diagnostic error is a concrete risk, and it determines an underestimation of the real number of affected subjects [13]. To date, more than 900 mutations in the GLA gene were described (Human Gene Mutation Database, www.hgmd.org), however not all these mutations are responsible for FD. A genetic variant can be unambiguously related to a disease and explain the phenotype of a patient, but not all genetic variants are involved in the patient’s symptomatology. Some variations may be only polymorphisms, individual variability, and these alterations could be interpreted, in certain cases, as a risk factor for the development of a disease. Fabry disease is suspected on the basis of clinical and anamnestic-familial data, and it is confirmed by genetic and enzymatic assays, such as the detection of the specific genetic alteration and the evaluation of α-Gal A activity, which could be null or deficient. Also, the determination of the enzyme substrates, Gb3 and its derivative globotriaosylsphingosine (LysoGb3), provides an important diagnostic support [14].

## 2. Results

**Figure 1:**
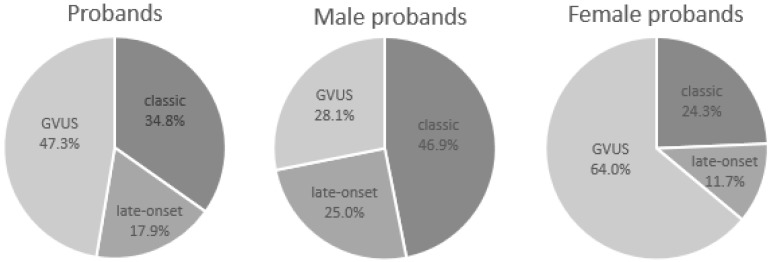
Phenotype observed in 207 Probands with a mutation in the galactosidase alpha gene (GLA).

Since 2005, our research group, operating at the Center for Research and Diagnosis on lysosomal storage diseases of the Institute of Biomedicine and Molecular Immunology of the National Research Council of Italy (IBIM-CNR) located in Palermo, has been devoted to the study of Fabry disease. In these years, in a case-finding project, we studied about 17,000 subjects (average age 47 years old, 55.9% males and 44.1% females, with average ages, respectively, of 47.9 and 45.2 years old) with clinical manifestations attributable to Fabry disease. In this population, we identified 207 Probands with a genetic alteration in coding regions or in regulatory portions (splicing sites) of the GLA gene (Table 1). In particular, in these subjects we detected 81 different mutations, 25 of which were described by us for the first time in the world (Table 2). Figure 1 shows the mutations, divided into classes according to the phenotype of patients in whom these mutations were identified. The study was extended to the relatives of these patients and identified another 264 subjects with mutation in the exons of GLA, thus reaching 471 individuals positive in the genetic test for FD (Table 3).

**Figure 2:**
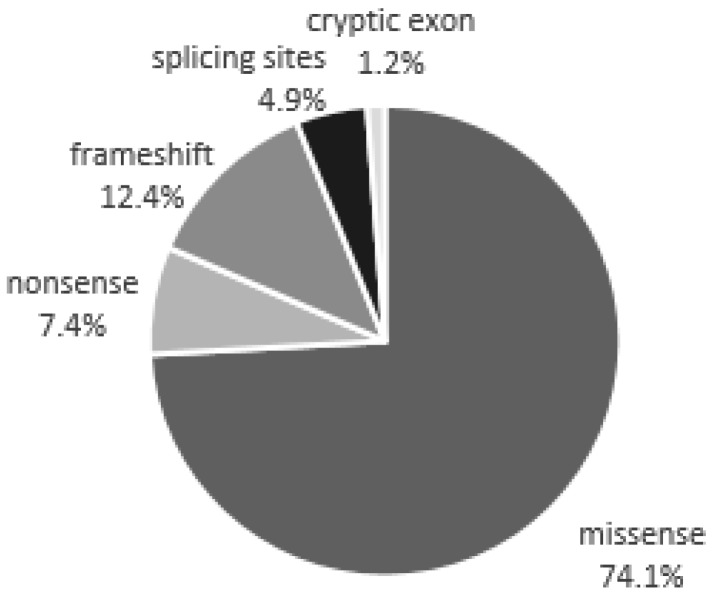
Classes of mutations that we found in the coding and regulatory regions of the GLA gene.

74.1% of the 81 mutations detected in our study are missense mutations, 12.4% are insertions or deletions of nucleotides that cause a frameshift during translation and the formation of a premature stop codon, 7.4% are nonsense mutations, 4.9% are mutations in the splicing sites involved in the regulation of mRNA maturation, and 1.2% are mutations in the cryptic exon of the gene (Figure 2).

71.6% of the 81 mutations detected were found in patients who had a classic phenotype of FD (58 mutations), 19.8% (16 mutations) were found in patients with late-onset phenotype and mild symptoms, and 8.6% (7 mutations) were found to be a genetic variant of unknown significance (GVUS) (Table 4). Regarding the 207 Probands, 34.8% of them (72 patients) had a classic phenotype, 17.9% (37 patients) had late-onset, and 47.3% (98 patients) had a GVUS (Table 4). Concerning the total of 471 subjects with a mutation in the GLA gene, 36.5% of them (172 subjects) had a mutation associated with classic phenotype, 23.6% (111 subjects) had a mutation associated with with late-onset, and 39.9% (188 subjects) had a GVUS (Table 4).

**Figure 3:**
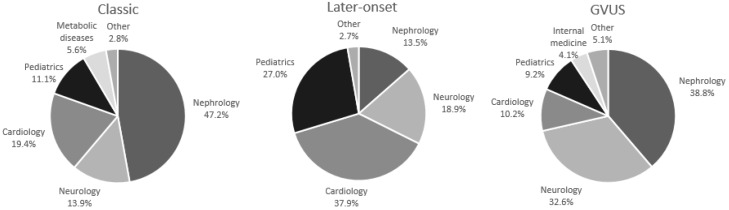
Clinical unit of origin of the Probands identified in our study.

In 47.2% of the 72 Probands with a classic phenotype, the diagnostic hypothesis of FD was advanced by a nephrologist, in 19.4% by a cardiologist, in 13.9% by a neurologist, in 11.1% by a pediatrician, in 5.6% from a metabolic diseases unit, and in 2.8% from another origin (Table 1 and Figure 3). In 37.9% of the 37 Probands with a late-onset phenotype, the diagnostic hypothesis of FD was advanced by a cardiologist, in 27% by a pediatrician in the context of neonatal screenings, in 18.9% by a neurologist, in 13.5% by a nephrologist, and in 2.7% from another origin (Table 1 and Figure 3). In 38.8% of the 98 Probands with a GVUS, the diagnostic hypothesis of FD was advanced by a nephrologist, in 32.6% by a neurologist, in 10.2% by a cardiologist, in 9.2% by a pediatrician, in 4.1% from a internal medicine unit, in 1% from a metabolic diseases unit, and in 4.1% from another origin (Table 1 and Figure 3).

**Figure 4:**
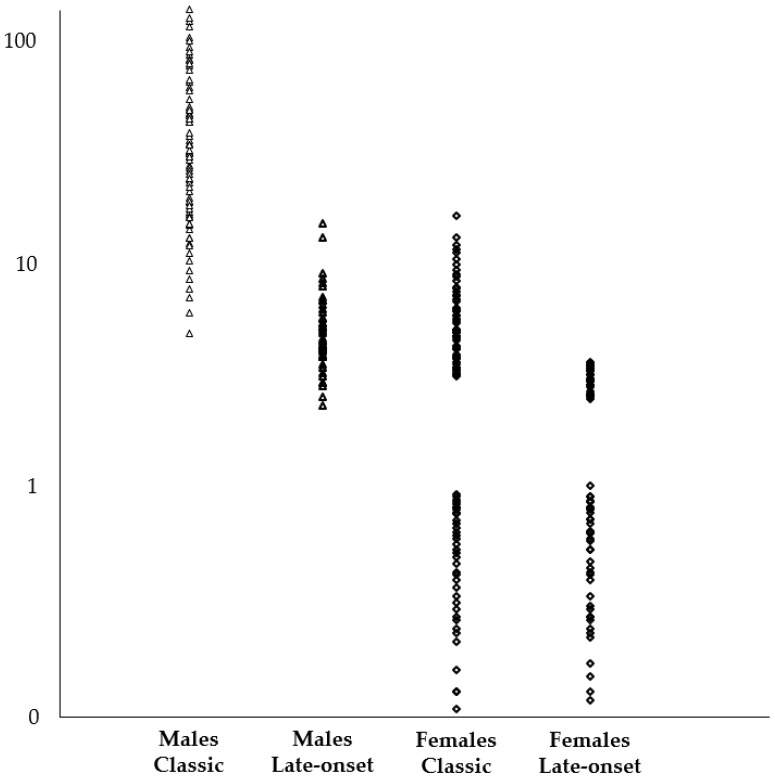
LysoGb3 concentration in blood of patients. LysoGb3 is measured in nmol/L; normal values 0.08–1.13 nmol/L. Logarithmic scale.

In all 125 male patients harbouring a mutation in the GLA gene that is associated with the classic or late-onset phenotype, the activity of the enzyme α-Gal A was always below normal values (Table 5). In the 158 female patients with the same mutations, the enzyme activity showed variability, with a range from pathologic to normal values (Table 5). In particular, only in 46% of female patients the enzyme activity was below normal values, while in 54% it was normal. Regarding the measurement of accumulation of LysoGb3 in blood, we found pathological values in all male patients with a mutation in the GLA gene causative of FD: the accumulation is remarkable (both in the average values and in the maximum value) in subjects with a mutation associated with classic phenotype, while it is modest in individuals with mutation associated with a late-onset variant (Table 5 and Figure 4). A blood accumulation of LysoGb3 was also found in 56.8% of female patients with a mutation in GLA associated with classic phenotype and in 40.1% of females with a mutation associated with late-onset variant; the values in both cases were lower than values found in males with the same type of mutations (Table 5 and Figure 4).

In all the samples that came to our attention, we also studied the intronic portions flanking the exons of the GLA gene. In 1.7% of the subjects positive in the genetic test, we found alterations in the splicing sites that were involved in the regulation of gene expression. All male patients studied by us with these mutations showed signs and symptoms attributable to the classic form of the disease, high levels of LysoGb3 in blood, and absent or strongly reduced α-Gal A activity. In four patients, we found the IVS4 + 919G > A mutation, already reported in the literature as associated with the late-onset, cardiac variant of FD [15].

We also found mutations in the non-coding regions of the GLA gene, located outside of splicing sites, most of which are grouped into haplotypes. The prevalence of these intronic mutations in patients with a symptomatology attributable to FD (28%) was approximately 2-fold higher than the prevalence observed in the control population (13%). All these patients have normal enzyme activity and no LysoGb3 accumulation in blood; therefore, they cannot be considered as being affected by FD. 

## 3. Discussion

The increase in population screening in recent years has led to the identification of a large number of genetic variants (mutations). A mutation can be clearly related to a disease and therefore explain the phenotype of a patient, but not all genetic variants are responsible or co-responsible for a particular disease or involved in patient’s symptomatology. The pathogenicity of a mutation and its relation to the clinical phenotype, in a patient, may remain unclear; some genetic variants can be polymorphisms, individual variability. These considerations are particularly important for many inherited disorders, including lysosomal storage disorders. In Fabry disease, to date more than 900 mutations in the GLA gene have been identified, but a percentage of these are classified by scientific community as genetic variants of unknown significance (GVUS). These mutations often pose a real problem with respect to counseling, family screening, and treatment options. Various studies have investigated FD in newborn screening and in screening of high-risk populations, to define appropriate criteria and parameters for a reliable FD diagnosis [16,17,18,19]. FD is suspected based on clinical and anamnestic-familial data and confirmed by the identification of a specific genetic alteration and by measuring α-galactosidase A activity, which could be null or deficient. Moreover, an important diagnostic support could be given by the determination of the enzyme substrates Gb3 and its derivative LysoGb3.

Our experience, based on about 17,000 subjects studied, shows that, in this relatively large and genetically well-characterized cohort of patients, LysoGb3 levels in blood were significantly higher in FD patients compared to healthy controls and to subjects with FD-like symptomatology, who were suffering from cardiac or renal damage with a different origin than FD. In particular, considering patients with a mutation in the GLA gene, LysoGb3 levels in blood were higher in patients with the classic phenotype than subjects with mild phenotype (male patients with late-onset variants and females). Subjects with a GVUS in the GLA gene had LysoGb3 levels in blood comparable to healthy controls. In patients’ relatives, LysoGb3 levels were generally similar in same-sex patients and always higher in males than in females. Blood levels of LysoGb3 are not closely related to age of patients, and higher values were found in patients with more severe clinical manifestations. Interestingly, in patients with classic phenotype, LysoGb3 levels in blood were generally higher among males with severe mutations, causing an incomplete synthesis of α-Gal A, such as frameshift or non-sense mutations, which are responsible for the formation of termination codons during translation. LysoGb3 levels in blood were also high in male patients with mutations in splicing sites, where regulation of gene expression at the post-transcriptional level occurs. In patients treated with the enzyme replacement therapy, LysoGb3 levels in blood were reduced, both in males and females. Female patients with FD usually have a milder and more variable symptomatology than male subjects; the activity of α-Gal A can result in the normal range, and a moderate accumulation of LysoGb3 in blood was present in 40–60% of them. This occurs because female patients are usually heterozygous, and they have organs that are chimeras of normal and affected cells, since, during embryonic development, one of the two X-chromosomes in each somatic cell becomes transcriptionally inactive (lyonization). Until some years ago, these patients were considered carriers, but this idea was revised, and today we are aware that they can develop organ damage and severe clinical manifestations as male patients [20].

The presence of LysoGb3 in patients with FD was reported for the first time by Aerts’ group in 2008 when, analyzing Gb3 and other metabolites in plasma of Fabry subjects, he discovered that LysoGb3 levels were markedly higher than those of Gb3 [21]. LysoGb3 is the deacylated form of Gb3, and its origin in Fabry patients is due to the activity of the lysosomal enzyme acid ceramidase, which converts the excess of Gb3 in LysoGb3. LysoGb3, unlike Gb3, is a soluble molecule and can be more easily excreted from cells [22]. The production of lyso-lipids as a system to decrease cellular accumulation seems to be a common strategy for various lysosomal storage diseases, and elevated lyso-lipid levels have also been found in Krabbe disease (galactosylsphingosine) and Gaucher disease (glucosylsphingosine) [23,24]. Further studies showed that the concentrations of LysoGb3 are particularly high in the liver and intestine. These observations led researchers to hypothesize the existence of a “secret road”: confronted with the inability to degrade Gb3, in FD patients the excess of Gb3 is converted into LysoGb3, which, once transported to the liver, is secreted from the body via bile [25]. The correlation between plasma levels of LysoGb3 and the manifestations of Fabry disease was established in several studies [26]. Furthermore, it is clear that in Fabry hemizygotes, both in humans and mice, plasma LysoGb3 is already high at birth. Conversely, in female hemizygotes, LysoGb3 is low at birth and increases gradually with age, and there seems to be a correlation with the symptomatology [21]. Other studies reported the direct involvement of LysoGb3 in the pathogenesis of Fabry disease, for its ability to establish inflammatory phenomena, and because LysoGb3 has been shown to promote smooth muscle cell proliferation, which may contribute to the increased intima-media thickening frequently observed in patients [27]. Other evidence suggesting a direct role of LysoGb3 in the pathogenesis of FD is the correlation between life-time exposure to plasma LysoGb3 and disease severity [26]. LysoGb3 is often used in patients’ follow-up for evaluating the efficacy of therapeutic treatment.

Our case-finding study emphasizes the importance of adding to diagnostic criteria for a FD diagnosis the determination of LysoGB3 in blood and/or accumulation of Gb3 detected in biopsies. This consideration is also applicable to screening studies, in which a significant number of subjects with a variant in the GLA gene harbor a GVUS, as observed by Van der Toll et al. [28].

## 4. Materials and Methods 

### 4.1. Patients

Peripheral blood was collected, using EDTA as an anticoagulant, from subjects with clinical manifestations related to FD symptomatology and from Fabry patients’ relatives. The study was approved by the Hospital Ethics Committee of the University of Palermo, and signed informed consent was obtained from all participants.

### 4.2. Genetic Analysis

DNA samples were isolated from whole blood by column extraction (QIAamp DNA Micro Kit, Qiagen, Germany), and their concentrations were determined using a spectrophotometer. Eight pairs of primers were designed for the analysis of eight target regions containing the seven exons of the GLA gene, including the regulatory sequences flanking them and the cryptic exon. PCR products were purified and sequenced to detect mutations using an automated DNA sequencer at BMR Genomics. 

### 4.3. α-Galactosidase A Activity Assay

α-galactosidase A activity was performed in all male patients and in female patients with a positive GLA gene test. α-Gal A analysis was determined by the Dried Blood Filter Paper test (DBFP) described by Chamoles et al. [29], with the following modifications: spot of 5.5 μL of blood in 6 mm diameter circle paper was placed into a 96-well plate suitable for fluorimetric assays and incubated for 16 h at 37 °C in a thermomixer; the reaction was terminated by the addition of 250 μL of 0.1 mol/L ethylenediamine (pH 11.4). The fluorescence of background, which is not due to the specific enzyme activity, was determined for each sample, performing another reaction in the presence of 0.15 mmol/L 1-deoxygalactonojirimycin (DGJ, the inhibitor of α-galactosidase A) in 0.15 mol/L citrate phosphate buffer (pH 4.4). This background was subtracted from the fluorescence of the sample. In each assay, we added positive and negative controls and a calibration curve with 4-methylumbelliferone.

### 4.4. LysoGb3 Determination

The determination of LysoGb3 in blood was performed as previously described by Polo et al. [30]. 

## 5. Conclusions

The significant number of patients studied in our case-finding project allowed us to observe that in FD patients there is a complete correspondence between symptomatology, the presence of mutations in the GLA gene, the activity of the enzyme α-galactosidase A, and the accumulation of LysoGB3 in blood and/or accumulation of Gb3 detected in biopsies. In these patients, clinical manifestations are severe in the absence of enzyme activity and in the presence of significant accumulation of Gb3/LysoGb3 (classic form); mild and/or organ-specific, in the presence of residual enzyme activity and modest accumulation (late-onset variants); and mild or absent, in the presence of normal enzyme activity and absence of substrates accumulation (GVUS). In our experience, LysoGB3 accumulation in blood can be considered a useful marker for FD, like the Gb3 detectable in biopsies but much less invasive. Today, we have elements to generate a reliable diagnostic algorithm (Table 6).

## Figures and Tables

**Table 1:** Characteristics of the 207 Probands identified and the clinical unit from which they came. GVUS: genetic variants of unknown significance. Enzyme activity is measured in nmol/mL/h; normal values > 3 nmol/mL/h. LysoGb3 is measured in nmol/L; normal values 0.08–1.13 nmol/L.

**Overall**	**Probands**	**Male Probands**	**Female Probands**
**Patients (%)**	207	96 (46.4%)	111 (53.6%)
**Average age [min-max]**	44.9 [0–85]	42.3 [0–69]	47.0 [4–85]
**Average α-Gal A activity**	4.7	1.8	7.2
**Average LysoGb3**	11.9	24.4	2.0
**Nephrology %**	37.2	38.6	36.1
**Neurology %**	23.7	13.5	32.4
**Cardiology %**	18.4	22.9	14.4
**Pediatrics %**	13.0	17.7	9.0
**Metabolic diseases %**	2.4	2.1	2.7
**Internal medicine %**	1.9	−	3.6
**Other %**	3.4	5.2	1.8
**Classic phenotype (%)**	72 (34.8%)	45 (46.9%)	27 (24.3%)
**Late-onset (%)**	37 (17.9%)	24 (25.0%)	13 (11.7%)
**GVUS (%)**	98 (47.3%)	27 (28.1%)	71 (64.0%)
**Classic Phenotype**	**Probands**	**Male Probands**	**Female Probands**
**Patients (%)**	72	45 (62.5%)	27 (37.5%)
**Average age [min-max]**	45.8 [0–73]	45.4 [0–57]	46.5 [18–73]
**Average α-Gal A activity**	1.8	0.1	4.7
**Average LysoGb3**	27.9	43.3	4.8
**Nephrology %**	47.2	48.9	44.5
**Neurology %**	13.9	11.1	18.5
**Cardiology %**	19.4	20.0	18.5
**Pediatrics %**	11.1	13.4	7.4
**Metabolic diseases %**	5.6	2.2	11.1
**Internal medicine %**	−	−	−
**Other %**	2.8	4.4	−
**Late-Onset**	**Probands**	**Male Probands**	**Female Probands**
**Patients (%)**	37	24 (64.9%)	13 (35.1%)
**Average age [min-max]**	45.0 [0–78]	44.7 [0–69]	45.5 [14–78]
**Average α-Gal A activity**	2.1	0.6	4.5
**Average LysoGb3**	4.7	5.5	3.1
**Nephrology %**	13.5	20.8	−
**Neurology %**	18.9	4.2	46.1
**Cardiology %**	37.9	41.6	30.8
**Pediatrics %**	27.0	29.2	23.1
**Metabolic diseases %**	−	−	−
**Internal medicine %**	−	−	−
**Other %**	2.7	4.2	−
**GVUS**	**Probands**	**Male Probands**	**Female Probands**
**Patients (%)**	98	27 (27.6%)	71 (72.4%)
**Average age [min-max]**	44.2 [1–85]	34.0 [1–58]	47.3 [4–85]
**Average α-Gal A activity**	8.2	6.2	9.0
**Average LysoGb3**	0.9	0.8	0.9
**Nephrology %**	38.8	37.1	39.4
**Neurology %**	32.6	25.9	35.2
**Cardiology %**	10.2	11.1	9.9
**Pediatrics %**	9.2	14.8	7.1
**Metabolic diseases %**	1.0	3.7	−
**Internal medicine %**	4.1	−	5.6
**Other %**	4.1	7.4	2.8

**Table 2:** Mutations detected in our study, divided by classes according to the phenotype observed in patients in whom they were identified. Mutations in bold were described by us for the first time worldwide.

**Mutations Detected in Patients with a Classic Phenotype**			
Missense	Nonsense	Frameshift	Splicing sites
p.M1T	**p.Q57X**	c.124_125delTA	IVS2−2>G
p.L54F	p.W162X	**c.428dupC**	IVS3+1G>A
p.E59K	p.R220X	**c.583insGAATA**	**IVS4+5G>T**
p.R112C	p.R227X	**c.614delC**	IVS5+1G>T
**p.G116A**	**p.E341X**	**c.618del10 + c.629insA**	
**p.Y152H**	**p.C382X**	**c.666delC**	
p.A156V		c.718_719delAA	
p.D165H		**c.846_847delTC**	
p.D170N		c.946delG	
p.C172Y		c.1163delTCC	
p.G183A			
p.G183S			
**p.Y184N**			
**p.Y222D**			
p.C223Y			
**p.R227L**			
**p.W236S**			
p.L243F			
**p.N249K**			
**p.G261C**			
p.P265L			
p.I270T			
p.L275H			
p.Q279H			
p.Q279K			
p.A288D			
**p.M290T**			
p.R301G			
p.R342Q			
p.I354K			
p.R356G			
p.R356Q			
p.R356W			
p.E358G			
p.G361R			
p.R363H			
p.G373S			
**p.T412P**			
**Mutations detected in patients with late-onset**			
Missense			Cryptic exon
p.L19P p.V22G p.D33Y			IVS4+919G>A
p.M51I			
p.I91T			
p.R112H			
p.F113L			
**p.K130E**			
P.I253T			
p.N215S			
**p.T246I**			
p.M290L			
p.R301Q			
**p.G395A**			
**p.V413A**			
**Genetic Variants of Unknown Significance**			
Missense			
**p.N5D**			
p.R118C			
p.R118H			
p.S126G			
p.A143T			
p.D313Y			
p.T385A			

**Table 3:** Characteristics of the 471 subjects with a genetic alteration in the GLA gene identified (Probands + relatives). GVUS, genetic variants of unknown significance. Enzyme activity is measured in nmol/mL/h; normal values > 3 nmol/mL/h. LysoGb3 is measured in nmol/L; normal values 0.08–1.13 nmol/L.

**Overall**	**Patients**	**Male Patients**	**Female Patients**
**Patients (%)**	471	185 (39.3%)	286 (60.7%)
**Average age [min-max]**	40.2 [0–85]	40.1 [0–73]	40.2 [1–85]
**Average α-Gal A activity**	4.1	1.9	5.6
**Average LysoGb3**	9.7	19.8	2.6
**Classic Phenotype**	**Patients**	**Male Patients**	**Female Patients**
**Patients (%)**	172	78 (45.3%)	94 (54.7%)
**Average age [min-max]**	38.2 [0–85]	39.3 [0–63]	37.3 [6–85]
**Average α-Gal A activity**	2.2	0.1	3.9
**Average LysoGb3**	20.3	38.0	4.6
**Late-Onset**	**Patients**	**Male Patients**	**Female Patients**
**Patients (%)**	111	47 (42.3%)	64 (57.7%)
**Average age [min-max]**	40.2 [0–78]	45.5 [0–73]	36.2 [6–78]
**Average α-Gal A activity**	2.4	0.5	3.7
**Average LysoGb3**	3.8	5.0	2.1
**GVUS**	**Patients**	**Male Patients**	**Female Patients**
**Patients (%)**	188	60 (31.9%)	128 (68.1%)
**Average age [min-max]**	41.9 [1–85]	36.7 [1–77]	44.2 [1–85]
**Average α-Gal A activity**	7.2	5.5	8.1
**Average LysoGb3**	0.9	0.8	0.9

**Table 4:** Phenotype associated with 81 mutations detected in individuals with a mutation in the GLA gene, phenotype of 207 Probands, and 471 positives (Probands + relatives)in genetic test.

	n. Mutations	% Mutations	n. Probands	% Probands	n. Positives	% Positives
**Classic**	58	71.6%	72	34.8%	172	36.5%
**Late-onset**	16	19.8%	37	17.9%	111	23.6%
**GVUS**	7	8.6%	98	47.3%	188	39.9%
**Overall**	**81**		**207**		**471**	

**Table 5:** Enzyme activity and blood accumulation of LysoGb3 in 471 patients studied.

		α-Gal A Activity	% Patients with LysoGb3 in Blood	Average LysoGb3 [min-max]
**Classic Phenotype**	**Males**	Null or almost null	100%	38.0 nmol/L [4.8–137.2]
	**Females**	Variable	56.8%	7.2 nmol/L [3.1–16.2]
**Late-onset**	**Males**	Residual	100%	5.0 nmol/L [2.3–15]
	**Females**	Variable	40.1%	3.2 nmol/L [2.5–3.6]
**GVUS**	**Males**	Normal	0	−
	**Females**	Normal	0	−
**Healthy controls**	**Males**	Normal	0	−
	**Females**	Normal	0	−

**Table 6:** Characteristics of subjects presenting a mutation in the GLA gene.

Mutation	Symptomatology	α-Gal A Activity	LysoGb3 in Blood
**Responsible for Classic Phenotype**	Systemic, severe	Null or almost null	Yes (high)
**Responsible for Late-Onset Phenotype**	Single organ and/or late-onset	Residual	Yes (low)
**GVUS**	Mild or absent	Normal	No

## Abbreviations

FD	Fabry Disease
α-Gal A	α-galactosidase A
GVUS	genetic variants of unknown significance
